# Volatile Organic Compounds in Indoor Air: Sampling, Determination, Sources, Health Risk, and Regulatory Insights

**DOI:** 10.3390/toxics13050344

**Published:** 2025-04-26

**Authors:** Tajana Horvat, Gordana Pehnec, Ivana Jakovljević

**Affiliations:** Institute for Medical Research and Occupational Health, 10000 Zagreb, Croatia; thorvat@imi.hr (T.H.); gpehnec@imi.hr (G.P.)

**Keywords:** VOCs, human health, air sampling, chromatography

## Abstract

Indoor air pollution is a serious public health issue caused by the accumulation of numerous toxic contaminants within enclosed spaces. Particulate matter (PM_2.5_ and PM_10_), biological contaminants (mould, bacteria, and allergies), inorganic gases (carbon monoxide, carbon dioxide, ozone, and nitrogen dioxide), and a variety of volatile organic compounds (VOCs) are examples of common indoor air pollutants. VOCs are one of the chief indoor contaminants, and their effects on human health have made indoor air quality a serious concern. Indoor VOC concentrations are frequently higher than outdoor levels, according to studies, which raises the danger of exposure, particularly for young people and those with respiratory disorders. VOCs originate from both biogenic and anthropogenic sources, and they can create secondary pollutants like ozone and aerosols, which can lead to cardiovascular and pulmonary problems. Prolonged exposure to VOCs has been associated with respiratory irritation, neurological effects, and an increased risk of chronic diseases. This review examines the primary sources, sampling and analysis approach, and health impact of VOCs in indoor air. Additionally, we compared worldwide regulatory guidelines for VOC exposure limits, emphasizing the need for strict exposure limits to protect human health.

## 1. Introduction

As people spend most of their time in indoor environments, investigations have focused on monitoring indoor air quality in the last two decades, especially due to the fact that concentrations of pollutants were, in general, found to be much higher indoors than outdoors, which can lead to multiple health outcomes such as respiratory and cardiovascular diseases [1]. Because of their long-term effects, even very low concentrations of pollutants in indoor air might pose a health risk [2]. According to the World Health Organization (WHO), more than three million people die annually from these diseases [3]. Volatile organic compounds (VOCs) are some of the most significant air pollutants of today due to their numerous sources and possible human health impacts. VOCs are, in fact, hundreds of gaseous organic trace species, which are directly emitted by biogenic and anthropogenic sources and are developed in the atmosphere. Also, VOCs were some of the most investigated pollutants in interior air [4]. They are considered precursors for forming secondary pollutants, including aerosols and ozone (O_3_) [5,6,7].

Indoor air is influenced by a variety of factors, including behavioural factors, environmental factors, dwelling characteristics, and indoor and outdoor pollution sources [8]. Indoor air pollution levels can be impacted by outdoor air pollution concentrations from both human and natural causes, including dust re-suspension, road traffic, industry, and wildfire smoke. Additional factors that affect the dispersion characteristics of pollutants around a building include topography, weather, the type of location, and distance of pollutant sources, as well as the size, shape, orientation, and arrangement of buildings [1,9].

Some EU member states have established guideline levels for key indoor air contaminants [10]. The European Coordinated Action on Urban Air, Indoor Environment, and Human Exposure has worked on harmonised emissions labelling under the Construction Product Directive (CPD) (89/106/EEC), with a focus on emissions that have an impact on indoor air [11]. The WHO, through the INDEX [12] and ENVIE projects [13], developed indoor air quality standards in 2010 to guide countries that lack legal regulations. In recent years, certain countries, such as Germany, Canada, Japan, and the Flemish Government, have established guidelines for indoor air contaminants and made significant progress in reducing indoor air pollution. While France and California utilise separate VOCs, the UK uses solely the total volatile organic compounds (TVOCs) as a metric. Germany and Canada, on the other hand, use a combination of these two [14].

The average exposure levels for the five VOCs (formaldehyde, benzene, toluene, trichloroethylene, and tetrachloroethylene) during different periods are specified in the Chinese national standard for indoor air quality (GB/T 18883) [15,16,17]. Table A1 presents the regulations and recommended limits for various VOCs in indoor air. Benzene, recognised worldwide as a carcinogen, has no safe level of exposure according to the WHO and South African guidelines. However, China has set a threshold of ≤0.03 mg m^−3^ for 1-h exposure. The European Union does not currently control the amount of these compounds in indoor air, mostly because there are insufficient data on hazard and risk assessments. On the other hand, the European Commission has made a substantial contribution to our understanding of how poor indoor air quality affects human health and well-being through its Environment and Health Strategy [18], Action Plan [19], and Health and Consumer Protection Strategy [20,21].

The main objective of this review paper is to consolidate the comprehensive knowledge of volatile organic compounds (VOCs) within a single document. To achieve this, the paper systematically examines the following: (1) the major indoor sources of VOCs; (2) a comparative analysis of active and passive sampling methodologies; (3) advanced analytical techniques employed for VOC quantification; (4) human exposure pathways and associated health effects; (5) reported concentration levels of VOCs in residential environments. A large literature search was conducted to identify any studies of volatile organic compounds in household air from 2010 to 2025. Only original research papers published in English in academic journals indexed in the Scopus index, Science Direct, Web of Sciences, and Google Scholar databases were used to collect data. The keywords used in searches, “Volatile Organic Compounds” OR “VOC” OR “VOCs”, resulted in 8313 papers, which was further filtered with the keywords “indoor air” OR “indoor air pollution”, resulted in 423 papers. Finally, the keywords “measurements” OR “monitoring” AND “home” OR “households” were used, which yielded a total of about 243 papers.

## 2. An Overview of VOC Determination: From Definition to Measurement Methodology

### 2.1. Definition and Sources of VOCs

According to the Environmental Protection Agency (EPA) VOCs are classified as compounds with a boiling point between 50 °C and 250 °C at a standard atmospheric pressure of 101.3 kPa. VOCs with molecular weights up to 150 g mol^−1^ are present in the gaseous phase at room temperature and can be detected in the air samples, while molecular weights up to 225 g mol^−1^ are semi-volatile or non-volatile at room temperature and cannot be detected in the air, they tend to adsorb to surfaces, dissolve in condensed water, or partition into aerosol particles, rather than existing freely in the gas phase [22,23].

Higher molecular weight VOC compounds cannot be detected in the air as they do not exist in the gas phase at room temperature. The WHO further classifies indoor organic pollutants as very volatile organic compounds (VVOCs), volatile organic compounds (VOCs), and semi-volatile organic compounds (SVOCs) (Table 1) [24]. VOCs include alcohols, aldehydes, hydrocarbons, ketones, organic acids, terpenoids, etc. [24]. BTX (benzene, toluene, xylene) are common VOC markers, with formaldehyde and acetaldehyde being major indoor carbonyl compounds [4]. Their sources include outdoor air, human activities (smoking, cooking, cleaning), and building materials [25]. In the monitoring of benzene, toluene, ethylbenzene, and *ortho*- (*o*-), *meta*- (*m*-), and *para*- (*p*-) xylenes (BTEX) in restricted industrial atmospheres, toluene was likewise the main chemical detected [26].

VOCs are ubiquitous in the environment, with sources of biogenic and anthropogenic origin. Plants, animals, and microorganisms can emit biogenic VOCs (BVOC) or they are the outcome of processes that include metabolism or decomposition. In contrast, anthropogenic VOCs (AVOC) originate from fossil fuel combustion in vehicles, solvents used for paints, adhesives, plastics, personal care and cleaning products, furniture and building materials, tobacco smoke and cooking. Phuc and Oanh [27] noted that the improved fuel quality used by vehicles significantly reduced VOC levels. The most prevalent BVOCs are terpenes (such as *α*-pinene, limonene), alcohols and carbonyls and from the anthropogenic group benzene, toluene, ethylbenzene, and *o*-, *m*-, and *p*-xylenes are the most researched VOCs, regarded as major indoor pollutants [24,28,29]. For BVOCs, it has been noted that they have higher reactivities towards oxidants compared to AVOCs [30,31,32], with over 70% of global BVOC emissions occurring in tropical regions, particularly from plant leaves [33,34].

Both BVOCs and AVOCs contribute to the photochemical production of secondary organic aerosol (SOA) and ground-level ozone when NOx and sunlight are present [35]. SOA are formed through the photochemical oxidation of VOCs and the condensation of subsequent low-volatility compounds [6]. Furthermore, BVOCs and AVOCs can interact and speed up the formation of SOA and O_3_ [36]. For almost all VOCs, the decomposition is initiated by a reaction with hydroxyl radicals to form hydro- or peroxy-radicals (HO_2_ or RO_2_). The main role of VOCs in the formation of the ozone layer is through radical reactions in which they consume NO or convert NO to NO_2_ as seen in Equations (1)–(3):

RH + OH• → HR• + H_2_O (1)

R• + O_2_ → R• + RO_2_• (2)

RO_2_• + NO → RO• + NO_2_ (3)

where RH• stands for radicals that form when an alkane RH loses a hydrogen atom. The way that VOCs can accelerate the rate at which NO is converted to NO_2_ and raise tropospheric O_3_ concentrations is succinctly explained by these equations [37,38,39]. The negative impacts that VOCs and NOx have on the environment and human health have led to their designation as the primary precursors of ozone production [40,41]. Reducing VOCs may not significantly alter tropospheric O_3_ concentrations. In the stratosphere, VOCs relevant to O_3_ depletion are commonly known as halogenated gases, including fluorine, chlorine, bromine, and iodine compounds. In the presence of shortwave radiation from the sun, halogenated gases release substantial amounts of stable halogens, destroying thousands of O_3_ molecules before being removed from the stratosphere [42]. However, alkenes and aromatics were major contributors to ground-level O_3_ production [43]. Additionally, higher temperatures were often linked to an increase in the O_3_ mixing ratio due to enhanced photochemical reactivity and source emission rates, while lower relative humidity was also connected to rising O_3_ mixing ratios [44].

### 2.2. Methodologies for Measuring VOCs in Indoor Air

Vera et al. [45] identified nine ISO standards for indoor sampling, and Table 2 presents four additional ISO standards. These standards offer guidelines for the quantitative assessment of VOC emissions, and understanding the contributions of their sources is vital for developing effective air pollution control strategies.

#### 2.2.1. Familiar Techniques for Sampling VOCs

**Figure 1:**
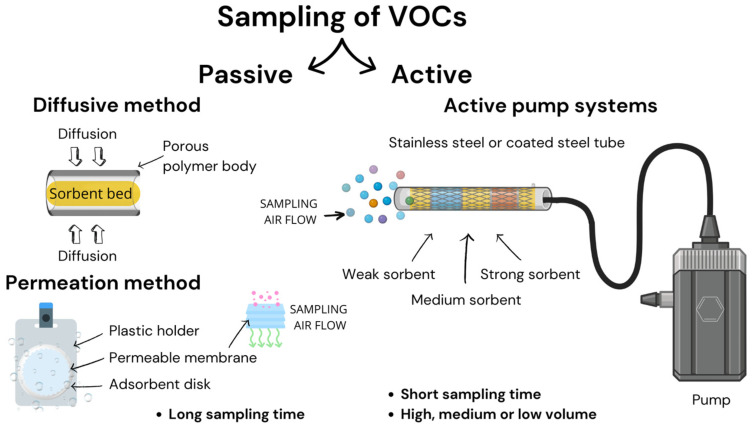
Types of sampling—active and passive.

Different techniques exist for measuring VOC concentrations, which some authors categorise as offline or manual techniques and online or automatic techniques [45]. In this work, a more straightforward classification was established between active and passive sampling, as well as for sensors. The analyte’s physical and chemical characteristics, anticipated levels, availability of electricity, spatial capacity, and the intended measurement (continuous monitoring, screening, personal/professional exposure, etc.) all influence the sampling approach used. Furthermore, sampling may be conducted using reactors, chambers, badges, bags and tubes (Figure 1). The geometry of tubes provides significant advantages over larger reactors, chambers, or rooms for studying the interactions between materials and VOCs. Tubes exhibit high experimental repeatability owing to their wall contact and reduced influence of convection and mixing compared to larger spaces [60,61,62,63,64,65].

##### Active Sampling

Active sampling uses pumps to draw a known volume of air through the sampling medium. The apparatus comprises an inlet, an intake tube designed to be as short as feasible and constructed from a material that minimises adsorption or retention of the analyte, an absorption medium, a flow regulator, an air flow meter, and a pump. The flow rate is usually in the millilitres per minute (e.g., between 10 and 250 mL min^−1^) range for gases and vapours and litres per minute for aerosols. Despite the fact that active sampling requires an electrical supply, most indoor air sampling pumps nowadays are battery-operated, enabling continuous sampling for more than twenty-four hours. Depending on the analyte being sampled, sampling can be performed in solution, on filter paper, or on an appropriate sorbent tube. The downside of the active sampling technique is that it is less user-friendly and more expensive than passive sampling due to the price of the pump, air flow meter, and air flow calibrator. Subsequently, the sample analysis is performed in the laboratory utilizing spectrometric or chromatographic methods. Active sampling has the advantages of allowing for more rapid sample collection, a controlled sample volume, and effective sampling of low concentrations of VOCs. Active sampling can be conducted using sorbent tubes, reactors, chambers, or canisters. Due to their geometry, sampling with tubes exhibits high experimental reproducibility.

##### Passive Sampling

Passive sampling collects pollutants in the air using absorption material without the use of a pump and works on the principle of free diffusion or diffusion through a semipermeable membrane or deposition. This sampling describes Fick’s first law using Equation (4):

(4) $$M=\frac{SA}{L}\times aCt$$

where M is the amount of analyte transported by diffusion, A is the diameter of the tube, S is the permeability coefficient (cm^2^ min^−1^), L is length (cm), C is analyte concentrations (mol cm^−3^), t is time (s), and a is constant [66,67].

Determining diffusive uptake rates (URs) is the most challenging step in passive sampling. Numerous factors influence uptake rates, including sorbent selection, tube configuration, duration of exposure, and environmental conditions. Automated thermal desorption (ATD) tubes provide exceptional flexibility for users in selecting sorbents and determining tube configurations based on the analytes being tested. However, they add complexity to the determination of URs. Various studies have derived URs from diffusion theory, conducted experiments in laboratory chambers, and compared active and passive sampling in the field. An effective UR (UR_eff_) is desirable because theoretically derived URs exhibit significant biases in real-world applications. Equations (5) and (6) demonstrate the performance of UR_eff_ compared to the ideal UR (UR_ideal_, in mL min^−1^) as follows:

(5) $${UR}_{ideal}=D\frac{A}{L}\times 60$$

(6) $${UR}_{eff}=\alpha {UR}_{ideal}$$

where D is the diffusion coefficient of compounds (cm^2^ s^−1^), A is the cross-sectional area of the tube (cm^2^), L is the air gap between the sampling end of the tube and the surface of the sorbent (cm), and 60 is for the conversion coefficient from mL s^−1^ to mL min^−1^. Further, UR_eff_ is a percentage of UR_ideal_, and this percentage can be defined as the sampling efficiency α, which indicates the sampling duration, e.g., 1 h or 7 days. Unfortunately, only a limited subset of VOCs has established and validated UR_eff_ values in the laboratory or in the field. This determination necessitates complex instrumentation, well-controlled laboratory environments, and long-term field tests, making it impractical for general users to conduct experimental determinations of URs [68,69,70,71].

Different chemical potentials or partition coefficients on either side of the barrier are the cause of molecular transport [72]. Diffusion barrier types can be divided into two categories: passive diffusion samplers and passive permeation samplers [73]. The advantages of this sampling method are easy performance, low price, and absolutely no need for electric energy, so it is possible to set it in different places simultaneously. On the other hand, disadvantages include a higher limit of detection, low precision, and the fact that it is usually not applicable for daily sampling, but it is applied for long-term periods [69,74].

##### Automatic Devices

Automatic devices utilise various measurement techniques that depend on pollution levels. Examples include spectrometric methods such as UV or IR absorption, fluorescence, or chemiluminescence. These techniques benefit information of the analyte levels directly, without sample handling, obtaining minute values, hourly averages, and online monitoring. Adverse circumstances involve complex and expensive equipment, the necessary electrical energy, and specific conditions, such as suitable locations and temperature conditions. They are large and sometimes difficult to install on-site, and they are also unsuitable for studying human exposure and routine research requiring multiple measurement sites. Regular maintenance of equipment is essential [75].

##### Sensor

Reliable data on indoor and outdoor air quality and conditions can be obtained thanks to the recently noted dynamic advancements in analysis techniques and instruments. However, this advancement raises the expense of air quality assessment and monitoring, which severely restricts their broad use. Therefore, a search for other ways to obtain information on air quality is underway. Sensor approaches are given special consideration. Sensors are set up to measure in hard-to-reach areas near pollution sources. They are devices that use long-path spectroscopic techniques to facilitate real-time monitoring of concentrations. The advantages of these sensors are low costs, sample design, and ease of use, while the disadvantages are excessively high limits of detection and quantification. Such sensors are nonselective and, therefore, mostly employed to assess the total VOC concentration, and they are unable to identify specific types of volatiles. However, some sensor systems are designed specifically for some targeted VOCs, such as formaldehyde or benzene. Table 3 shows commercially available sensor systems used for determination of VOCs.

#### 2.2.2. Types of Sorbents Used for Sampling VOCs

**Figure 2:**
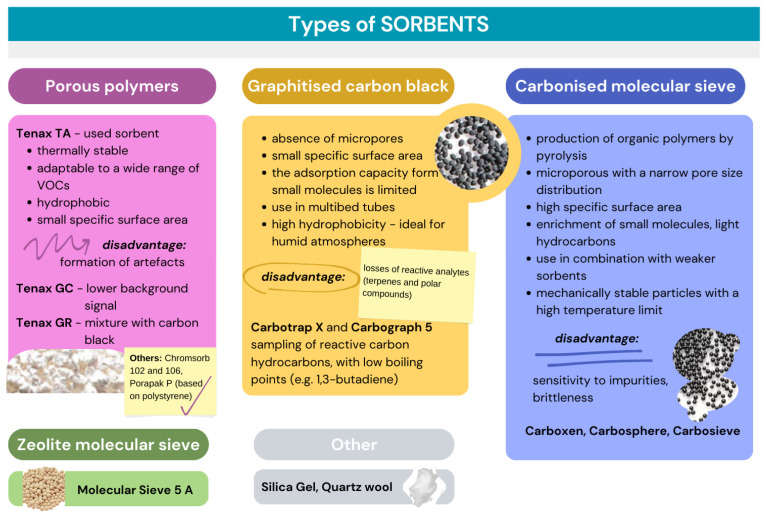
The main features of different types of sorbents.

Figure 2 illustrates their main characteristics. Among these, the most commonly utilised sorbents are those from the group of porous polymers and graphitised carbon black, often employed in mixtures. Regarding standardisation, the ISO 16000-6:2021 standard specifies a method in which Tenax TA, Carbopack X, or Carbograph 5TD may serve as sorbents [51]. Additionally, it is important to note that tubes containing sorbents like Tenax TA can generate low levels of benzene artefacts over time, even though it has been shown that these tubes do not contain benzene immediately following conditioning. False positive results can significantly affect the determination of benzene at low concentrations. Therefore, it is recommended that any low benzene values be verified through independent testing using a tube filled with an alternative sorbent, such as carbon black. This is one of the main reasons why a mixture of several sorbents is selected for sorption [85]. As early as 1984, the US EPA’s TO-2 method proposed using a sorbent based on carbon molecular sieves instead of Tenax TA for specific compounds with a boiling point between 15 and 120 °C [86]. Subsequently, two methods published by the same organisations, TO-15 and TO-17, suggest using stronger sorbents for VOCs while also addressing the issue of water retention [87,88].

As previously mentioned, Tenax TA poly-(2,6-diphenyl-p-phenylene oxide) is the most widely used sorbent due to its high thermal stability (up to 350 °C), hydrophobicity and versatility with a broad spectrum of volatile compounds [69]. Variants include Tenax GC (lower background signals) and Tenax GR (mixed with graphitised carbon black). Due to its modest specific surface area (30 m^2^ g^−1^), it is primarily suited for sampling compounds with medium to high boiling points, such as hydrocarbons from -C_6_ at room temperature. Furthermore, the degradation of reactive analytes, including terpenes, may occur, especially in the presence of ozone within the sample. Other porous polymer sorbents include Chromosorb 101, 102, 106, Porapack P, HayeSep P, and XAD-2, all polystyrene-based materials. They are less thermally stable and not ideal for thermal desorption. Chromosorb 106 is the most frequently used material; however, it can produce elevated background levels, making it impractical for trace analysis. The selected sorbents should be capable of capturing and desorbing the desired analytes with low artefact rates. Porous polymers are characterised by low adsorption of very volatile organic compounds as they pass through the sorbent. Graphitised carbon blacks expand the range of analytes, while carbon molecular sieves can absorb even smaller polar molecules [89].

Graphitised carbon blacks are highly hydrophobic sorbents suitable for low-vapour-pressure compounds like 1,3-butadiene. To analyse a wide range of compounds, multibed tubes can be used, which are mixtures of up to three different sorbents arranged in order of increasing strength. After preparing the multibed tubes, they should be stored appropriately to prevent irreversible adsorption of less volatile compounds onto the stronger sorbents [51]. The second group of significant sorbents consists of graphitised carbon blacks, characterised by the absence of micropores. Consequently, their specific surface area is small (5–260 m^2^ g^−1^), and their adsorption capacity for small molecules. It has been established that the higher the degree of graphitisation, the smaller the specific surface area of the final material. These sorbents are frequently employed in multibed tubes, and due to their high hydrophobicity, they serve as ideal sorbents for sampling in highly humid atmospheres. The carbon molecular sieves make up a third important group of sorbents, which are produced by the pyrolysis of organic polymers. Microporous materials have a narrow pore size distribution and high specific surface areas (250–400 m^2^ g^−1^). They are designed to enrich small molecules, such as light hydrocarbons. Carbon molecular sieves are mechanically stable particles with a high-temperature limit (>400 °C) and are commercially available under the trade names Carboxen, Carbosphere, Carbosieve, or Ambersorb [90,91]. It has been reported that a combination of various sorbents, including Tenax GR, Carboxex, and Carbosieve SIII, is necessary to capture a broad range of VOCs, particularly under humid sampling conditions, while achieving greater desorption efficiency [89,92].

Samples collected with sorbent tubes can be directly injected into a Gas Chromatograph (GC) via thermal desorption (TD) or extracted by solvent extraction (SE) or headspace (HS), which will be discussed in the following subsection.

#### 2.2.3. Analytical Method for VOC Determination

Many VOCs are in very low concentrations in environmental samples and, therefore, difficult to detect without the use of highly sensitive analytical techniques. Furthermore, matrix interferences may make measurement much more difficult.

The literature reveals that gas chromatography is predominantly used to identify VOCs in the air, alongside the solvent extraction (SE) method and solvent-free methods for sample preparation. Solvent-free methods include thermal desorption (TD), solid-phase microextraction (SPME), and needle trap devices (NTDs). SPME and TD can be used for sampling VOCs from bags or other vials with samples of different matrices (e.g., soil, blood, urine, food, and beverages) [93,94,95,96,97,98]. The SE and SPME methods are simpler than the TD method because they require no special equipment and have been used for many years in various sample preparation applications [34,99,100]. SPME sorbents, such as divinylbenzene and polyacrylate, cannot be strictly regarded as adsorbents, as the analytes are absorbed through distribution within the polymer material rather than by surface adsorption. Nevertheless, thermal desorption provides greater sensitivity for analysis than solvent desorption, but a single-sample analysis cannot be replicated. Nowadays, the new model of thermal desorption allows the re-collection of split samples during thermal desorption, but a short investigation showed that the efficiency of the re-analysis of re-collected samples was 50.0 ± 5.6% of the total amount at second cycles [101]. This method is a fully automated, solvent-free method for high-sensitivity gas extraction, requiring minimal manual preparation and reducing analyst error. The process involves heating the sample or sorbent in a helium or nitrogen carrier gas stream to desorb volatile and semi-volatile analytes for GC analysis. Key parameters of thermal desorption include the desorption temperature, carrier gas flow rate, desorption time, and selection of the sorbent that serves as the stationary phase. TD offers a higher recovery rate of 95%, compared to traditional solvent extraction (~75%). Furthermore, common interference issues associated with solvent extractions include masking peaks of interest, signal suppression of solvent-eluting components, and baseline distortion. Additionally, depending on the volatile compounds of interest, thermal desorption enables the selective purification of sample interferences, such as water or ethanol, before analysis. Conversely, many extraction solvents, such as carbon disulphide (CS_2_), are toxic and odorous, presenting significant potential health and safety risks. In this respect, thermal desorption is regarded as safer and can be installed without the need for additional fume hoods; however, its drawback is the high initial cost [63,102,103]. The sampling and analysis methods mostly used in the determination of VOC are shown in Table 4.

Table 4 shows that liquid chromatography is also used alongside GC to measure VOCs in air, and various detectors are listed. The studies employed mass spectrometry as the most common detector, a flame ionisation detector (FID), and a photoionisation detector (PID). The main difference between them is their level of sensitivity [113]. Liquid chromatography is occasionally used for the analysis of thermally unstable, highly polar, or water-soluble VOCs, such as formaldehyde, phenols, alcohols, and organic acids that are difficult to analyse by GC. The literature discusses liquid chromatography equipped with a fluorescence detector (FLD) and a diode array detector (DAD) for determining VOCs. VOCs are separated in the liquid phase utilizing a high-pressure pump, a stationary phase (e.g., a C_18_ column), and a mobile phase. The most common detection methods are mass spectrometry (MS), fluorescence, and ultraviolet-visible spectroscopy (UV-Vis). Derivatisation with 2,4-dinitrophenylhydrazine (DNPH) improves detection sensitivity for carbonyl substances such as formaldehyde.

When comparing GC and LC techniques for VOC determination, GC proved more sensitive than LC, although LC had faster analysis time [112]. The high-performance liquid chromatography (HPLC) technique uses methanol and water as the mobile phase, while helium is the carrier gas in the GC technique. Furthermore, when comparing various detectors in gas chromatography, the MS detector was found to be the most suitable for determining lower quantification limits than the FID, PID and electron capture detector (ECD). One drawback of the MS detector is its inability to distinguish between *meta*- and *para*-xylene, resulting in their combined quantification. In contrast, due to its polar column, the PID detector enables the separation of xylene isomers [114]. A comparison of HPLC and GC methods for the determination of VOCs is shown in Table 5.

However, an enhanced analytical method for comprehensively characterising VOCs in indoor air involves thermal desorption–2D GC–MS (TD-GC × GC-MS). This technique separates VOCs with similar structures before detection, such as monoterpenes and sesquiterpenes. Consequently, cleaner spectra are obtained, facilitating library searches and spectrum interpretation, ultimately leading to a more thorough characterisation of VOCs in indoor air [115].

### 2.3. Human Exposure Assessment

The US EPA classified some VOCs, such as acrolein, acrylamide, acrylonitrile, benzene, vinyl chloride, ethylene oxide, (ethyl)benzene, 1-bromopropane, 1,3-butadiene, carbon disulphide, propylene oxide, styrene, tetrachloroethylene, toluene, trichloroethylene, and xylene, as hazardous air pollutants [116]. Numerous VOCs have the potential to cause cancer, mutagenesis, genotoxicity, and neurotoxicity. Research has indicated that human exposure to VOCs raises the risk of respiratory disorders, leukaemia [117,118], birth abnormalities [119], neurocognitive impairment [120], dermatitis [121] and cancer [122]. Human exposure to VOCs can occur through inhalation [123], ingestion [124] and dermal contact [125], while inhalation is the dominant route of exposure to VOCs. The International Agency for Research on Cancer (IARC) has classified VOCs such as benzene as carcinogenic (Group 1), while others like ethylbenzene are possible carcinogens (Group 2B), and toluene and xylene are considered non-carcinogenic (Group 3) (Table 6) [107]. Indoor air pollution is especially significant for vulnerable groups, such as children aged 0 to 16, who tend to spend extended periods indoors [126]. A child’s immune and respiratory systems are still developing, making them more susceptible to airborne environmental pollutants. As a result, most research on VOC exposure has been conducted primarily in kindergartens, preschools, and schools.

Most of the studies have assessed non-cancer inhalation risk using the methodology proposed by Liu et al. [14] and the US EPA [127].

Non-cancer inhalation risk was assessed by calculating the hazard quotient (HQ), as detailed in Equation (7):

(7) $$HQ=\frac{E}{RfC}$$

where *HQ* is the hazard quotient, *E* is the exposure level, and *RfC* is the inhalation reference concentration for chronic non-cancer health effects (mg m^−3^). Then, exposure levels of VOCs can be estimated according to Equation (8):

(8) $$E=C\times \frac{ET}{24}\times \frac{EF\times ED}{AT}$$

where *E* is the exposure level, *C* is the concentration of VOCs (µg m^−3^), *ET* is exposure time (hours day^−1^), *EF* is exposure frequency (days year^−1^), *ED* is exposure duration (years), *AT* is averaging time (years). Lifetime cancer risk (*LCR*) can be calculated by multiplying the exposure levels and inhalation unit risk of a specific VOC (Equation (9)). The IUR for specific VOCs is shown in Table 6 [14,127].

(9) LCR=E×IUR

According to categorisations of lifetime cancer risks, LCR > 1.0 × 10^−4^ is taken to be a definite risk, and a possible risk is when LCR is higher than 1.0 × 10^−5^ and lower than 1.0 × 10^−6^.

**Table 6:** Inhalation unit risk (IUR) (m^3^ μg^−1^) for specific VOCs [128].

VOC	IARC Group	IUR
**Benzene**	1	2.9 × 10^−5^
**Toluene**	3	n.a.
**Xylenes**	3	n.a.
**Acetaldehyde**	2B	2.7 × 10^−6^
**1,4-dichlorobenzene**	2B	1.1 × 10^−5^
**1,3-Butadiene**	2A	1.7 × 10^−4^
**Trichloroethylene**	2A	2.0 × 10^−6^
**Tetrachloroethylene**	2A	6.1 × 10^−6^
**Methylene chloride**	2B	1.0 × 10^−6^
**Chloroform**	2B	5.3 × 10^−6^
**Ethylbenzene**	2B	2.5 × 10^−6^
**Naphthalene**	2B	3.4 × 10^−5^
**Formaldehyde**	2A	6.0 × 10^−6^

n.a.—not available.

The common VOCs present indoors are BTEX produced during fuel combustion, cigarette smoke and house renovations. Natural gas produces alkanes, 1,4-dichlorobenzene and a-pinene origin from moth repellents and wood-based building materials, respectively, whereas cleaning products produce limonene. The health effects and sources of indoor air VOCs are shown in Table 7. Formaldehyde, benzene and dichlorbenzene are associated with leukaemia. Some VOCs have a significant risk of eye and nose irritations (ether and aldehydes). Many VOCs have strong associations with headache (Table 7).

BVOCs may have both beneficial and detrimental effects on human health. BVOCs, such as those found in flowers and essential oils at specific concentrations, may have therapeutic properties that elevate mood and lessen stress. But when BVOCs react with air pollutants like nitrogen oxides (NOₓ), they create SOA and ground-level ozone, which can deteriorate air quality and cause lung irritation and asthma. Monoterpenes, a class of BVOC, are one of the most common VOCs found in indoor air. Insects and plants, especially conifers, pine trees, and eucalyptus, are the main producers of monoterpenes [129,130]. They are released by a variety of indoor objects, including wooden furniture [130]. However, some BVOCs at high concentrations can negatively affect animals and humans. For instance, 3-camphene, a monoterpene, may exacerbate allergic skin reactions and induce bronchoconstriction [131]. Monoterpenes readily undergo chemical reactions with airborne oxidative agents, such as OH•, NO_3_, or ozone, producing dangerous secondary pollutants such as organic acids, formaldehyde, and SOA [129]. Long-term indoor exposure to high BVOC levels can result in allergic responses, headaches, lightheadedness, and respiratory irritation. The harmful effects of indoor BVOCs on health can be lessened by the use of low-emission materials and proper ventilation.

**Table 7:** Indoor air VOCs and their health impact and possible sources.

VOCs	Possible Source	Health Impact	Ref.
ethanol, methanol, 2-propanol, acetone	Nail polish remover, cleaning products, hair sprays, perfumes	Headache, giddiness, insomnia, irreversible visual impairment or blindness, stomach disturbances, vomiting	[132,133]
formaldehyde, hexanal	Plywoods, carpets, furniture	Eyes irritation, cancer, leukaemia, coughing	[134,135]
ethylene, isobutylene	Rubber items	Headache, dizziness	[135]
benzene, toluene, ethylbenzene, xylene, dichlorobenzene, naphthalene, styrene	Deodorisers, tobacco smoke, air fresheners, foam products, furniture	Acute myeloid leukaemia, speech difficulties, headaches, dizziness, sleep disturbances, nausea	[136,137]
methyl-tert-butyl ether	Plasticisers, medical solvent	Nose irritation, headache, eye irritation, inability to coordinate	[137]
n-butane, n-hexane, n-heptane, n-octane, cyclohexane	Furniture, leather, cosmetics	Skin rash, muscular weakness, confusion, dizziness	[138,139]
methylene chloride, 1,1,1 trichloroethane, difluoromethane, tetrachloroethylene	Freezers, refrigerators, air conditioners	Headache, vomiting, convulsions, death	[140]
acetone, methyl ethyl ketone, methyl isobutyl ketone	PVC cement and primer, adhesives	Brain fog, headache, fatigue	[141]
α-pinene, D-limonene	Wood, citrus oil cleaners, cosmetics	Kidney damage, cancer, liver damage	[28,142]

According to Bentayeb et al. [143], there is a link between aromatic chemicals like toluene and *o*-xylene and the asthmatic symptom of nocturnal dyspnea in the elderly. Paciência et al. [144] found that a rise in the number of overweight and obese children was associated with higher levels of toluene and xylenes. Additionally, children had a much higher incidence of nasal congestion. The most carcinogenic VOCs are benzene, 1,4-dichlorobenzene, 1,3-butadiene, chloroform, and naphthalene (Table 6).

LCR values exceeding 1.0 × 10^−4^ for benzene and 1,3-butadiene for adults were found in Chinese residential houses [14], which is considered a definite risk. Values between 1.0 × 10^−5^ and 1.0 × 10^−4^, classified as probable risks, were obtained for benzene for male and female adults in houses in India [145] for indoor air measured in bedrooms in China [146], for children’s rooms in Argentina [147], as well as for adults in a non-smoker’s home in Hong Kong [148]. Also, these LCR values were obtained for acetaldehyde and formaldehyde for adults in living rooms in Spain and male and female adults at renovated houses in Taiwan, respectively [149,150]. Values below 1.0 × 10^−6^ were deemed negligible risks and were determined for benzene, tetrachloroethylene, 1,1-dichloroethane, methylene chloride, trichloroethene, tetrachloroethene, for adults in Turkey, China, Hong Kong, and India [14,145,148,151]. Table 8 shows the LCR values determined for indoor air around the world and in different house areas.

### 2.4. Concentrations of VOCs Measured in Households Throughout Europe and Around the World

Indoor VOC levels depend on many factors, such as the strength of emission sources, ventilation rates, and the indoor oxidative environment. These factors reflect differences in chemical use, building design and materials, occupant behaviour, and seasonal change [1,152,153]. Table 9 shows several studies that measured household VOCs in Europe and the rest of the world over the last ten years. In all of the households investigated, the indoor air concentrations of VOCs are expressed in μg m^−3^. The sampling methodologies differed across studies, making it challenging to compare the results clearly. In some instances, the sampling period lasted one month [150,154,155,156,157], in others, several months [145,146,158,159,160,161,162,163,164], and in a few cases, even several years [165,166]. Moreover, sampling predominantly took place in the living room, kitchen, and bedroom. Several studies also examined the relationship between indoor and outdoor environments, while two studies highlighted changes occurring between the winter and summer seasons [159,166]. In addition to the most studied VOCs like BTEX, the majority of research also examined styrene, terpenes, formaldehyde, and tetrachloroethylene. Table 9 illustrates that the concentrations of ethylbenzene, benzene, and heptane in households across various countries have decreased since 2019. Furthermore, other VOCs, such as tetrachloroethylene and trimethylbenzene, have recently been detected.

Some studies indicate that VOC concentrations are typically higher indoors than outdoors [167], emphasising the importance of assessing and exploring their impact on human health. This is due to the presence of indoor emission sources (e.g., paint and related supplies, household furnishings, consumer products, combustion appliances, etc.), low ventilation rates, environmental tobacco smoke, internal fuel combustion for cooking, seasonal variations in temperature and relative humidity, and various other environmental conditions. Also, the US EPA has reported that indoor VOC levels are approximately 2.5 times higher than outdoor levels. 

Gabriel et al. [158] asserted that most VOCs detected in households were attributed to emissions from indoor sources, such as building materials and detergents (notably terpenes) and high ratios of indoor-to-outdoor (I/O) concentrations were observed. However, significant correlations between indoor and outdoor concentrations were found as well, indicating that these compounds originate from similar sources. The same paper illustrates, as an example, the relationship between 1,2,4-trimethylbenzene and ethylbenzene, as well as the connection between ethylbenzene and benzene. Additionally, the mean indoor TVOC concentrations were significantly higher than those measured outdoors, recorded at 269.3 μg m^−3^ compared to 60.6 μg m^−3^ [158]. Indoor VOC concentrations were significantly higher in households that had been renovated in previous years or had poor ventilation [14]. However, Otgonbyamba et al. [162] observed lower concentrations of benzene, toluene, and formaldehyde in new buildings compared to older ones, while the levels of *m*-, *p*-xylene showed the opposite trend. It is commonly believed that relocating to a residential space more than a year after renovation and improving ventilation could considerably reduce indoor exposure to volatile organic compounds.

Caron-Beaudoin et al. [164] revealed that chloroform levels were higher in homes where smoking was allowed. Sources of chloroform in indoor air can vary, including drinking water that contains chloroform [168,169]. This study indicated that chloroform concentrations in indoor air and drinking water were correlated with a Spearman’s rank correlation coefficient of 0.520. Nearby industrial activities, such as hazardous waste disposal sites, were also mentioned as potential contributors to chloroform in indoor air. Similarly elevated values for chloroform were also recorded for benzene, ethylbenzene, *o*-xylene, tetrachloroethylene, and 1,2,4-trimethylbenzene, while significantly higher values were noted for limonene. Alvarez-Vaca et al. [165] noted how the heating system affects indoor VOC levels. Rural regions of the country that primarily use heating oil showed the highest average values of aromatic hydrocarbons, whereas urban areas using natural gas revealed the lowest average values. Heeley-Hill et al. [159] found a strong correlation between BTEX compounds that align with their common source, while weaker correlations were observed for terpenes. The behaviour of terpenes was monitored throughout seasonal distributions in both indoor and outdoor environments. Indoor concentrations of α-pinene were predominant in summer, whereas limonene was more prevalent in winter compared to outdoor levels. Alves et al. [154] measured VOC levels in a kitchen over two months. They found that indoor VOC concentrations did not correlate with the corresponding outdoor levels, indicating that the sources of emissions in kitchens differed from those encountered outdoors. Kozielska et al. [160] compared VOCs in offices, apartments, and residential buildings, noting higher concentrations in residential buildings. Mečiarova et al. [155] and Rodrigues Dos Santos et al. [161] presented values for TVOC, with the latter relating TVOC concentrations to episodes of wheezing and emphasizing that in rooms with low levels of TVOC, there were fewer episodes of wheezing in children [161]. Mečiarova et al. [155] also highlighted higher concentrations in family homes with attached garages, a point echoed by Yang [156], who noted that ventilation had a significant impact on VOC levels. Utilising mechanical ventilation can achieve lower levels than natural ventilation. Rovelli et al. [170] conducted sampling during the dishwasher washing cycle and, as expected, recorded concentrations of d-limonene that were two to three times higher than those of other VOCs. Similarly, Rösch et al. [163] observed the highest levels of terpenes in his research. Cheng et al. [146], Kumar et al. [145] and Thian et al. [171] compared BTX levels across various household rooms (the bedroom, living room, kitchen and hall room), noting the highest concentrations of toluene in all areas. Cheng et al. [146] further investigated formaldehyde, for which he also found elevated concentrations. Villanueva et al. [150] measured terpene concentrations, such as limonene and pinene, which were noted to be at higher levels. Uchiyama et al. [166] illustrated a seasonal distribution during the winter and summer months, revealing higher values during winter for all compounds except for toluene. In measuring VOC levels in newly renovated homes, Du et al. [157] indicated that elevated indoor levels of BTEX in recently renovated residences were predominantly attributed to indoor sources.

On the other hand, office environments have higher levels of benzene, ethylbenzene, *p*, *m*, *o*-xylenes and toluene than the other microenvironments. The kindergartens had the lowest concentrations of these VOCs; for instance, the frequency of *p*, *m*-xylenes was 47% in kindergartens and 69% in offices [172]. According to Wallenus et al. [172], concentrations of TVOC in public buildings did not exceed the reference value set by the Finnish Decree on Housing Health 545/2015 (400 µg m^−3^), so they considered that indoor concentrations of TVOCs in Finnish public buildings are generally low and pose no health risks.

Given all of the above stated, it is essential to emphasise the need for a standardised approach to data collection, sampling methodologies, and a clear reporting system to ensure comparability. The next chapter delves into VOCs in greater detail, highlighting their behaviour and key characteristics.

**Table 9:** Concentrations of VOCs in households from previous investigations.

Author	City, Country	Sampling Time	Description	Compounds, μg m^−3^	Ref.
Thiam et al., 2025 ^a^	Senegal	July–September 2020; October–December 2021;	bedroom (n = 15), living room (n = 21), hall room (n = 22)	Benzene 7.2 (b), 6.9 (l), 12.6 (h) Toluene 10.2 (b), 18.3 (l), 80.6 (h)	[171]
Gabriel et al., 2024 ^a^	Northern Portugal	July 2018–June 2019	bedroom, living room n = 30	Benzene 1.2, ethylbenzene 1.8, *m*/*p*/*o*-xylenes 15.4, styrene 1.0, tetrachloroethylene 0.3, toluene 15.5, 1,2,4-trimethylbenzene 2.0, 3-carene 1.7, *α*,*β*-pinenes 8.6, limonene 18.2, TVOC 269.3	[158]
Otgonvbyamba et al., 2023 ^b^	Ulaanbaatar, Mongolia	November 2019–July 2020	new buildings n = 380 old apartments n = 144	Benzene 15.2, *m/p*-xylenes 82.9, toluene 68.1, formaldehyde 5.2 for new buildings Benzene 24.2, *m/p*-xylenes 71.8, toluene 74.9, formaldehyde 20.4 for old apartment	[162]
Caron-Beaudoin et al., 2022 ^c^	Peace River Valley, Canada	May–September 2019	family room or bedroom	Benzene 0.80, ethylbenzene 0.80, chloroform 0.80, cyclohexane 0.20, heptane 1.10, *m*/*p*-xylenes 3.00, *o*-xylene 0.90, styrene 0.90, tetrachloroethylene 0.20, toluene 4.10, 1,2,4-trimethylbenzene 0.60, limonene 28.2	[164]
Alvarez-Vaca et al., 2022 ^b^	Grand Duchy, Luxembourg	2014–2019	living room, kitchen, office	<p75: benzene 2.7 (n = 369), formaldehyde 11.9 (n = 345), *β*-pinene 3.5 (n = 370), limonene 10.3 (n = 370) <p90: ethylbenzene 2.1 (n = 370), heptane 2.7 (n = 370), *m*-xylene 3.8 (n = 370), *o*-xylene 2.2 (n = 370), tetrachloroethylene 0.2 (n = 370), toluene 8.7 (n = 370), 1,2,3-trimethylbenzene 0.9 (n = 370), 1,2,4-trimethylbenzene 2.9 (n = 370), 1,3,5-trimethylbenzene 0.8 (n = 370), 1,4-dichlorobenzene 0.3 (n = 370) Maximum value: *p*-xylene 1.8 (n = 370), styrene 1.7 (n = 370), trichloroethylene 0.3 (n = 370), 3-carene 1.7 (n = 370), *α*-pinene 4.9 (n = 370)	[165]
Heeley-Hill et al., 2021 ^c^	Ashford, United Kingdom	February–April July–September 2019	winter–summer n = 60	Benzene 0.50, ethylbenzene 0.80, n-heptane 0.30, 2-methylpentane 0.40, *m/p*-xylenes 1.50, *o*-xylene 1.20, tetrachloroethylene 0.03, toluene 1.50, dichloromethane 0.20, 1,3,5-trimethylbenzene 0.20, *α*-pinene 8.00, *β*-pinene 0.1 limonene 3.80	[159]
Alves et al., 2020 ^a^	Aveiro, Portugal	October–November 2017	kitchen	Benzene 1.6, ethylbenzene 2.4, *m/p*-xylenes 7.6, *o*-xylene 3.1, styrene 1.0, tetrachloroethylene 0.96, trichloroethylene <0.10, toluene 9.4, 1,4-dichlorobenzene <0.10, *α*-pinene 9.5	[154]
Kozielska et al., 2020	Upper Silesia, Poland	February–May 2017	residential building and flats, weekend	Benzene 3.59, ethylbenzene 5.41, *m/p*-xylenes 3.88, *o*-xylene 0.78, styrene 5.20, toluene 15.70, 1,3,5-trimethylbenzene 4.52 r. buildings Benzene 1.07, ethylbenzene 1.35, *m/p*-xylenes 0.68, *o*-xylene 1.72, styrene 2.61, toluene 16.45, 1,3,5-trimethylbenzene 3.10 flats	[160]
Mečiarova et al., 2017 ^d^	Slovakia	May 2017	living room	519.7	[155]
Rodrigues Dos Santos et al., 2020 ^a,c^	Lisabon, Portugal	October 2015–March 2016	bedroom	TVOC 0.2 ^c^ TVOC 3.1 ^a^	[161]
Yang et al., 2020 ^a^	Switzerland	September 2015	bedroom	Benzene 4.1, n-heptane 9.0, xylenes 22, toluene 51, formaldehyde 14, *α*-pinene 4.5, d-limonene 14, TVOC 384	[156]
Rovelli et al., 2019 ^a^	Como, Italy		during a dishwasher washing cycle, n = 9	Benzene 4.0, ethylbenzene 4.8, *m*-xylene 13.8, *p*-xylene 4.3, *o*-xylene 4.9, styrene 0.9, toluene 23.4, *α*-pinene 6.2, d-limonene 231.5	[170]
Cheng et al., 2018 ^a^	Chongqing, China	November 2014–February 2015	bedroom, living room, kitchen	Benzene 6.6 (b), 9.1 (l), 11.6 (k) Xylene 14.3 (b), 14.2 (l), 15.2 (k) Toluene 24.4 (b), 23.6 (l), 21.4 (k) Formaldehyde 23.2 (b), 21.3 (l), 15.6 (k)	[146]
Cheng et al., 2016 ^a^	Melbourne, Australia	August 28 to December 2008 Januray 12 to May 4 2009	living room	Methylcyclopentane 0.8, cyclohexane 1.3, n-heptane 1.8, methlycyclohexane 1.1, benzene 1.3, toluene 10.7, ethlybenzene 1.2, *p*-xylene 2.9, *m*-xylene 1.2, styrene 0.5, *o*-xylene 2.2, 1,3,5-trimethylbenzene 0.8, 1,4-dichlorobenzene 0.2, α-pinene 5.8, β-pinene 3.9, d-limonene 11.3	[173]
Villanueva et al., 2015 ^a^	Puertollano, Spain	May–June 2011	living room	Benzene 1.9, ethylbenzene 3.4, n-heptane 3.2, *m/p*-xylenes 7.0, *o*-xylene 0.8, styrene 2.1, toluene 12.0, 1,2,4-trimethylbenzene 2.9, formaldehyde 54.6, *α*-pinene 18.5, d-limonene 17.1	[150]
Uchiyama et al., 2015 ^a^	Japan	winter/summer January–March July–September 2012, 2013, 2014	residential buildings n = 602	Benzene 2.3/1.3, ethylbenzene 5.6/4.4, *m+p*-xylenes 8.3/5.8, *o*-xylene 3.4/2.6, toluene 11.0/12.0, 1,3,5-trimethylbenzene 2.0/1.2	[166]
Kumar et al., 2014 ^a^	New Delhi, India	March–May 2011	residential homes, kitchen, living room, bedroom	Benzene 7.9 (k), 8.2 (l), 7.3 (b) *m+p*-xylenes 4.2 (k), 5.4 (l), 4.7 (b) *o*-xylene 1.8 (k), 2.3 (l), 1.9 (b) toluene 30.7 (k), 32.9 (l), 28.8 (b)	[145]
Rösch et al., 2014 ^a^	Leipizig, Germany	May 2006–December 2008	n = 662	Benzene 1.5., cyclohexane 1.60, chlorobenzene 2.93, ethylbenzene 1.51, heptane 4.20, methylcyclopentane 1.31, *m/p*-xylenes 3.27, *o*-xylene 0.97, styrene 0.83, trichloroethylene 0.17, toluene 13.18, 1,3,5-trimethylbenzene 0.41, 1,2,4-trimethylbenzene 1.35, 1,2,3-trimethylbenzene 0.40, *α*-pinene 31.69, *β*-pinene 3.69, *σ*-3-carene 15.54, limonene 28.31	[163]
Du et al., 2014 ^c^	Guangzhou, China	December 2014	newly renovated homes	Benzene 18.8, *m+p*-xylenes 46.0, *o*-xylene 33.9, toluene 181.0	[157]

n—number of households; ^a^ mean; ^b^ geometric mean; ^c^ median; ^d^ average.

## 3. Discussion

### 3.1. Impact of Human Activities on Indoor VOC Levels

#### 3.1.1. Cooking’s Impact on VOC Levels

Cooking activities have been found to influence the levels of VOCs present in indoor air. Cooking temperature has been reported to positively correlate with organic pollutants, including particulate matter and volatile organic compounds. VOCs were measured to reach their highest levels approximately five minutes after cooking ended, while particulate matter took around 15 min to attain peak levels. Their physical properties can explain this phenomenon, as VOCs exist in the gaseous phase at room temperature, and gases disperse more rapidly than particulate matter [5]. The VOC concentrations in homes using various cooking fuels were compared. The levels of 1,2,4-trimethylbenzene, styrene, toluene, and heptane in homes that use gas were significantly higher than in those that use electricity, as these VOCs are either impurity components of cooking fuel or by-products of combustion [174]. A comparison of cooking with liquefied petroleum gas (LPG) and natural gas revealed that LPG adversely affects indoor VOC levels. Using natural gas as an alternative cooking fuel can improve kitchen air quality compared to other fossil fuels [175].

#### 3.1.2. Renovation’s Impact on VOC Levels

The next significant activity affecting VOC levels is renovation. Finishing paints, soft flooring, and final touches represent the phases with the highest recorded VOC concentrations. Measurements have indicated that the use of finishing paints is associated with elevated levels of aldehydes, while wood-based panels serve as a notable source of formaldehyde [176]. Furthermore, formaldehyde levels were particularly high in newly constructed homes furnished with recently purchased items [1,126,177,178]. According to the WHO (2010) [8], the formaldehyde levels for indoor air quality in Europe are set at 100 µg m^−3^ as a 30-min average. As temperatures rise, formaldehyde is released in greater amounts, indicating a strong correlation with indoor temperature and humidity. It is widely recognised that VOC concentrations in households are influenced by temperature, humidity, and the rate of air exchange [179]. Due to their high mobility, VOCs are also noted for their ability to penetrate materials and diffuse into buildings. According to Fang et al. [180], humidity has a substantial impact on chemical emissions from waterborne floor varnish and wall paint. Rising moisture in the air may cause more hydrophobic VOCs to be extracted from the material surface, increasing the rate at which those chemicals are released [180]. The release of VOCs from furniture and building materials can be accelerated by high humidity. Moisture can degrade adhesives and surface coatings, increasing the amount of formaldehyde and other compounds that are released into the air. Chlorophenols, which were once employed as wood preservatives in construction materials, are mostly generated by microbial methylation. Even at extremely low concentrations, it is strongly odorous and can generate major indoor air quality complaints despite the fact that they are not acutely hazardous at ordinary exposure levels [181].

High VOC concentrations have frequently been observed in newly constructed or renovated residential buildings [182,183,184], with new buildings generally incorporating built-in mechanical ventilation that lowers TVOC concentrations [156]. 

#### 3.1.3. Impact of Cleaning Products on VOC Levels

Cleaning household surfaces with products that contain alcohols, terpenes, glycol ethers, and chlorinated solvents can increase VOC concentrations in indoor environments. A huge group of cleaning products contains ethylene glycol ethers as solvents. EPA classified glycol ethers as hazardous air pollutants. Also, possible causes for concern are terpene hydrocarbons and terpene alcohols, frequently generated from plant oil. Terpenes are used as active solvents in some cleaning products and perfumed agents in consumer products. These substances produce formaldehyde, hydrogen peroxide, hydroxyl radical and SOA in reaction with ozone [185,186,187]. According to Bello et al. [188], ten-minute cleaning sessions with glass and bathroom cleaners raised overall VOC concentrations for up to 20 min after the cleaning operation ended. Singer et al. [189] investigated concentrations of limonene and showed that it can be found in 10 to hundreds of milligrams per cubic meter in air and persist for many hours after cleaning.

### 3.2. Concentration of VOCs in Different Types of Rooms and Ventilation Systems

Some authors have reported higher VOC concentrations in basements than on upper floors, attributing them to vehicle emissions from a garage within the house. Attached garages are known to influence indoor levels of VOCs, such as benzene and ethylbenzene [126,156,190]. Furthermore, indoor VOC concentrations increased with the number of occupants, including both people and pets, correlated with higher emissions from respiration and metabolic processes [174]. Concentrations also varied according to occupants’ daily activities, such as cooking, cleaning, and smoking [191]. Heating with a wood burner or open space heater raises BTEX emissions in the room [190]. Levels in the kitchen and living room are often compared, with slightly lower concentrations reported in the living room [175,192]. Larger houses with more rooms tend to have lower VOC concentrations [193].

Various studies have demonstrated a clear relationship between building type and VOC concentrations, which are higher in private homes than in public buildings or workplaces. This can be explained by differences in building materials and ventilation systems, among other factors [194]. Indoor air ventilation systems can significantly affect air quality. Several studies have highlighted increased levels of VOC concentrations in areas with inadequate ventilation. Numerous investigations into ventilation systems have been conducted in environments frequented by children, such as school classrooms [195]. Regular natural ventilation of classrooms by opening the windows reduced total indoor VOC levels [196]. Monitoring the TVOC concentration before and after the ventilation improvement showed a decrease from 71.5 to 10 µg m^−3^ [197]. Indoor environments with natural ventilation exhibited higher mean VOC concentrations compared to those with mechanical ventilation, indicating the efficacy of mechanical systems in reducing indoor air pollutants [198,199]. For example, in indoor environments with natural ventilation, formaldehyde, toluene, xylenes, and d-limonene levels were 15 µg m^−3^, 26 µg m^−3^, 5.8 µg m^−3^, and 11 µg m^−3^, respectively, compared to environments with mechanical ventilation where concentration levels of these pollutants were 13 µg m^−3^, 16 µg m^−3^, 1.4 µg m^−3^, and 7.9 µg m^−3^, respectively [156]. Generally, a smaller area is naturally ventilated in winter than in summer. This leads to the assumption that the difference in VOC concentration levels between naturally and mechanically ventilated areas is even more pronounced in winter due to the reduced dilution of pollutants in the air of naturally ventilated areas [200]. Additionally, the contribution of outdoor air to indoor air will depend on the ventilation system, indicating that ventilation will directly influence the air exchange rates with outdoor air [201]. Increased VOC levels indoors during winter months are frequently linked to insufficient ventilation during this season [126].

### 3.3. Indoor vs. Outdoor VOC Levels

Concentrations of most VOCs indoors are found to be higher than those outdoors [150,202]. This is particularly evident during the summer periods when high temperatures and increased relative humidity are observed. TVOC indoor concentrations increased significantly when indoor temperatures surpassed 30 degrees, and relative humidity exceeded 60 per cent [167,203]. Also, carpets, fabrics, and drywalls can absorb VOCs at low humidity and re-release them at higher humidity, causing fluctuating indoor VOC levels [204]. Fang et al. [180] investigated emissions of VOC from carpets, polyvinyl chloride (PVC) flooring, sealant, floor varnish, and wall paint within a relative humidity range of 30–70%. They found that, with increasing humidity, the concentrations of TVOC for wall paint and floor varnish increased dramatically. However, the impact of humidity was not noticeable for carpets, PVC flooring, or sealant. Besis et al. [205] correlated the increase in indoor summer concentrations, in contrast to colder periods, with the greater number of cars and ships arriving at this summer destination, as elevated concentrations were also noted in the outdoor air. In contrast, lower concentrations of benzene and terpenes, such as alpha-pinene and d-limonene, were recorded in summer compared to winter. During winter, terpenes are less involved in reactions with ozone and are observed in higher concentrations [206]. Kozielska et al. [109] found that benzene concentrations were twice as high in winter as in summer. The elevated benzene levels were attributed to heating systems combined with emissions from motor vehicles. It is clear that indoor VOC levels are influenced by outdoor air. In summer, when solar radiation is at its peak, the variation in outdoor VOC concentrations is affected by both their photooxidation reactions with the hydroxyl radical and their emissions from various sources. In contrast, during winter, when the rate of these photooxidation reactions is at its lowest, changes in VOC concentrations are more influenced by variations in emissions from sources rather than by photochemistry. It has been reported previously that the lifetime of VOCs in the troposphere is 20 times shorter in summer than in winter, considering the OH• radical reaction as the rate-determining step in the photooxidative degradation of VOCs [39,40]. During summer, photochemical reactions of VOCs are enhanced and consequently contribute significantly to the processes of VOC production and loss, depending on the rate constant of each VOC. Kumari et al. [207] also reported lower BTEX levels in winter, particularly in January, due to sampling being conducted after rainy days. Consequently, the reduced levels were attributed to BTEX’s high solubility in water [208]. It has been observed that the direction and speed of the wind may also influence VOC levels. At higher wind speeds, the concentration of VOCs decreases due to the dispersion of these compounds. For benzene, higher concentrations were noted when wind speeds were low and blowing from the southeast-southwest direction [209]. It is generally understood that over time, relatively low levels of solar radiation result in reduced VOC oxidation mediated by hydroxyl radicals (OH•), which ultimately leads to the accumulation of VOCs in the lower layers of the atmosphere. Meteorological conditions, including temperature, wind speed and direction, are significant factors associated with air pollution episodes [210]

Akteruzzaman et al. [192] revealed that VOC levels also decreased from morning to evening. In Jin et al. [211], the concentrations of TVOCs in the afternoon (205 µg m^−3^) were comparable to those in the morning (200 µg m^−3^), indicating consistent VOC emissions in indoor air throughout the day. By examining the behaviour of VOC levels on weekdays and weekends, it was found that concentrations decreased over the weekends [212,213,214]. Vallecillos et al. [214] observed a reduction in ethanol concentration from 306.04 µg m^−3^ during the week to 37.99 µg m^−3^ over the weekend. Additionally, Parra et al. [213] monitored BTX and noted a similar decrease in concentrations during the weekend, which was attributed to reduced traffic at the time.

Despite seasonal variations, certain VOCs exhibited similar correlations during both summer and winter. For instance, xylenes are typically strongly correlated with toluene. This correlation arises from their similar lifetimes in the troposphere and shared primary emission sources. The long lifetimes of benzene and toluene, which are approximately 12 and 2 days, respectively, contrast with xylenes, which have a lifetime of about 7 h due to their higher atmospheric reactivity and earlier release [215]. This difference may explain their higher ambient air concentrations and, consequently, those found indoors [207]. Among the BTEX compounds, the concentrations of xylene and ethylbenzene are lower than those of benzene and toluene, a phenomenon attributed to their more photochemically reactive characteristics compared to benzene and toluene. Notably, toluene is incorporated into fuels to enhance octane ratings, thereby contributing to its concentration [207,216]. In contrast, styrene and n-nonane are examples of VOCs that poorly correlate during the summer campaign [217]. Terpenes exhibit high correlations as they originate from common internal sources, with a mutual correlation noted between chloroform and d-limonene [202]. Terpenes (α-pinene, camphene, limonene) represent the most prevalent group of compounds found in schools and kindergartens due to the frequency of their use as cleaning agents in these settings [218].

Spearman’s correlation coefficient is frequently utilised to identify common sources of VOC emissions. The indoor/outdoor ratio is typically employed to assess the influence of indoor environments and sources. An I/O ratio of <1 indicates that outdoor air quality primarily affects indoor air quality, whereas an I/O ratio of ≈1 suggests that indoor sources impact indoor air quality to a similar degree. In contrast, an I/O ratio of >1 signifies that predominantly endogenous emission sources, such as building materials, paints, varnishes, cosmetics, and electronic devices, are responsible for indoor air quality [150,219,220].

### 3.4. Spatial Distribution

Indoor VOC levels were significantly higher than outdoor levels in both rural and urban areas, regardless of traffic density [168]. VOC concentrations in indoor air in urban houses were found to be more polluted than in rural ones [1]. A comparison of schools in rural, urban, and industrial locations revealed the highest concentrations. The presence of benzene in schools located in industrial areas can be attributed to traffic and the petrochemical industry [203,221]. A study examining indoor and outdoor benzene concentrations in European cities during the period from 1996 to 2000 reported elevated levels in Mediterranean cities, contrasting with much lower levels in northern cities [222,223]. This difference can be explained by the higher temperatures that lead to greater evaporation and the type of ventilation employed; mechanical ventilation is more prevalent in northern Europe, while natural ventilation predominates in the southern regions of Europe [4].

## 4. Conclusions

This work outlines the differences in the behaviour of volatile organic compounds in indoor and outdoor environments and their possible anthropogenic and biogenic sources. It also notes that human activity significantly impacts the quality of both outdoor and indoor air. Everyday activities, such as cooking and cleaning, can lead to transient emissions of specific volatile compounds, including terpenoids and chlorinated compounds. Similarly, indoor and outdoor renovations encourage more stable emissions of these compounds from building materials and furniture. Given this context, there is a need for direct research on the short-term and long-term retention of VOCs in indoor air, particularly since people today spend most of their time indoors. A literature review indicates that studies on indoor spaces, such as schools, kindergartens, and workplaces, exist, while less information is available regarding VOC levels in residential buildings. Since the presence of VOCs in indoor environments can pose a potential health risk due to long-term human exposure, additional emphasis should be placed on such research. The consequences of prolonged human exposure range from mild irritation to severe chronic diseases, including respiratory disorders and carcinogenic effects. Studies have shown that some VOC levels have recently decreased, comparisons remain challenging due to variations in sampling and analysis techniques. Among the sampling techniques, passive sampling using Radiello attachments and active sampling utilizing pumps with a fixed flow rate and tubes filled with various sorbents are noteworthy. When selecting a specific sampling method, it’s essential to ensure that the sampling period adequately represents indoor air quality. Clear guidelines based on specific standards enhance the representativeness of volatile organic compound sampling. Still, further efforts are needed to develop standardised guidelines that can improve indoor air quality and mitigate potential health risks.

## Figures and Tables

**Table 1:** Classification of volatile organic pollutants [24].

Description	Boiling Point Range (°C)	Example Compounds
Very volatile organic compounds (VVOCs)	<0 to 50–100	Butane, propane, methyl chloride
Volatile organic compounds (VOCs)	50–100 to 240–260	Formaldehyde, terpenes, BTEX
Semi-volatile organic compounds (SVOCs)	240–260 to 380–400	Pesticides (DDT, phthalates), fire retardants (PCBs, PBB, PAH)

**Table 2:** The most important ISO standards for sampling and measuring VOC in indoor air.

ISO Standard	Scope	Ref.
ISO 16000-1:2004. Indoor air—Part 1: General aspects of sampling strategy	The sampling strategy for indoor air monitoring offers suggestions on the purpose, when, where, how often, and for how long monitoring is to be performed to develop suitable sampling.	[46]
ISO 16000-2:2004. Indoor air—Part 2: Sampling strategy for formaldehyde	The sampling strategy for accurately planning measurements of indoor formaldehyde pollution.	[47]
ISO 16000-3:2022. Indoor air—Part 3: Determination of formaldehyde and other carbonyl compounds in indoor air and test chamber air—Active sampling method	The document specifies the determination of formaldehyde (HCHO) and other carbonyl compounds in the approximate concentration range of 1 μg/m^3^ to 1 mg/m^3^. The document also outlines the subsequent analysis of the hydrazones formed by high-performance liquid chromatography (HPLC) with detection by ultraviolet absorption.	[48]
ISO 16000-4:2011. Indoor air—Part 4: Determination of formaldehyde—Diffusive sampling method	This part of ISO 16000 specifies the determination of formaldehyde in indoor air using a diffusive sampler with solvent desorption and HPLC. It indicates a range from 0.001 mg/m^3^ to 1.0 mg/m^3^ for a sampling period of between 24 h and 72 h.	[49]
ISO 16000-5:2007. Indoor air—Part 5: Sampling strategy for volatile organic compounds	This document aims to assist in planning measurements of indoor pollution from VOCs.	[50]
ISO 16000-6:2021. Indoor air—Part 6: Determination of organic compounds (VVOC, VOC, SVOC) in indoor and test chamber air by active sampling on sorbent tubes, thermal desorption and gas chromatography using MS or MS FID	This document outlines a method for determining the emissions of VOCs from products or materials used in indoor environments using test chambers and test cells. It applies to measurements at concentrations ranging from micrograms to several milligrams per cubic metre.	[51]
ISO 16000-9:2024. Indoor air—Part 9: Determination of the emission of volatile organic compounds from building products and furnishing—Emission test chamber method	This document specifies a general laboratory test method for determining the area-specific emission rate of VOCs from samples of newly produced building products or furnishings under defined climatic conditions.	[52]
ISO 16000-11:2024. Indoor air—Part 11: Determination of the emission of volatile organic compounds from building products and furnishing—Sampling storage of samples and preparation of test specimens	This document specifies the sampling procedures, transport conditions, storage, and substrates that can influence the emissions of volatile organic compounds for three types of building products or furnishings: solid, liquid, and combined.	[53]
ISO 16000-29:2014. Indoor air—Part 29: Test methods for VOC detectors	This part defines performance test procedures for VOC detectors referred to for monitoring indoor and living atmosphere VOC concentrations and controlling indoor air quality in portable, mobile, and remote applications.	[54]
ISO 16000-32:2014. Indoor air—Part 32: Investigation of buildings for the occurrence of pollutants	This part of ISO 16000 serves as a foundation for subsequent sampling of suspect areas and the determination of the type and quantity of pollutants, which are detailed in other sections of ISO 16000.	[55]
ISO 16000-33:2024. Indoor air—Part 33: Determination of phthalates with gas chromatography/mass spectrometry (GC/MS)	The document provides details on the sampling and analysis of phthalates in indoor air, dust, and solvent wipe samples from the surface, utilising GC-MS.	[56]
ISO 16000-41:2023. Indoor air—Part 41: Assessment and classification	The document specifies a procedure for assessing indoor air quality that is applicable to all interior spaces in both residential and non-residential buildings with natural or mechanical ventilation, where people occupy these spaces for more than a temporary period.	[57]
ISO 16017-1:2000. Indoor, ambient and workplace air—Sampling and analysis of volatile organic compounds by sorbent tube/thermal desorption/capillary gas chromatography—Part 1: Pumped sampling	This part of ISO 16017 provides general guidance for the sampling and analysis of VOCs in air. It applies to ambient, indoor, and workplace environments and the assessment of emissions for materials in both small and full-scale test chambers. The measurement of airborne vapours of VOCs is applicable within a concentration range of approximately 0.5 mg/m^3^ to 100 mg/m^3^ for individual compounds.	[58]
ISO 16017-2:2003. Indoor, ambient and workplace air—Sampling and analysis of volatile organic compounds by sorbent tube/thermal desorption/capillary gas chromatography—Part 2: Diffusive sampling	Part 2 of the ISO 16017 standard also provides guidance like part 1 for the sampling and analysis of VOCs in the air. However, it is also intended for measuring airborne vapours of VOCs within a mass concentration range of approximately 0.002 mg/m^3^ to 100 mg/m^3^ for individual organics with an exposure time of 8 h, or 0.3 µg/m^3^ to 300 µg/m^3^ for individual organics over an exposure time of four weeks.	[59]

**Table 3:** Sensors used for VOC determination.

Sensor	Measuring Technique	VOCs	Ref.
Optical	change in light parameters	benzene, butane, chlorobenzene, chloroform, dichloromethane, ethanol, ethyl acetate, formaldehyde, hexane, isopropanol, methane, methanol, propane, tetrahydrofuran, toluene, xylene	[76]
Surface Acoustic Wave	Frequency	ethanol, octane, toluene	[77]
Chemoresistors—Polymers	Conductivity	acetone, acetonitrile, benzene, butylamine, cyclohexane, ethanol, hexane, isopropanol, methanol, methylene chloride, toluene, xylenes	[78]
Chemoresistors—Graphene	Conductivity	acetone, benzene, chloroform, ethanol, hexane, isopropanol, methanol, propanol, trichloroethylene, toluene, *m*-xylene	[79]
Quartz Microbalance	Mass change	acetone, acetonitrile, ethanol, 3-methyl-1-butanol, 1-octanol, toluene, *p*-xylene	[80,81]
Field Effect Transistor (FET)	Voltage change	hexane, hexanol, hexylamine, naphthalene, trimethylamine	[82,83]
Nondispersive Infrared	Infrared-radiation absorption	infrared absorbing VOC’s (methane)	[84]

**Table 4:** Some examples of approaches to sampling and analysis methods in determining VOCs in the indoor environment.

Analysis Method	Column	Sampling Method	Duration	Sorbent Type	Ref.
SPME-GC-FID	CP-WAX 52CB (50 m × 0.32 mm, 1.2 µm) (*Agilent Technologies, Santa Clara, CA, USA*)	Passive	24 h	SPME fibre coating with mixture of Tetra-nbuthylorthototitanat and graphite	[104]
SPME-GC-MS	BP-20 (30 m × 0.53 mm, 0.50 µm) (*SGE Analytical Science, Ringwood, VIC, Australia*)	Passive	30 min	Carboxen Polydimethylsiloxane	[105]
GC-FID	HP-INNOWAX (30 m × 0.5 mm, 0.25 µm) (*Agilent Technologies, Santa Clara, CA, USA*)	Passive	28 days	Activated Charcoal	[106]
GC-MS	DB-624 (60 m × 0.25 mm, 1.4 µm) (*Agilent Technologies, Santa Clara, CA, USA*)	Passive	22 days	Activated carbon	[107]
NTD-GC-FID	Rt-TCEP (60 m, 0.25 mm, 0.4 mm) (*Restek, Bellefonte, PA, USA*)	Active	5 min	Polydimethylsiloxane Carbopack-X Carboxen-1000	[108]
TD-GC-FID	RTX-5 (30 m × 0.32 mm, 3.00 μm) (*Restek, Bellefonte, PA, USA*)	Passive	30 days	Tenax GR	[109]
TD-GC-MS	DB-5MS (30 m × 0.25 mm I.D.) (*Agilent Technologies, Santa Clara, CA, USA*)	Active	6 h	Tenax-TA Carboxen 1000 Carbosieve	[110]
HPLC-DAD	Synergi 4 μ Hydro-RP (15 cm length, 5 μm, 4.6 mm i.d.) (*Phenomenex, Torrance, CA, USA*)	Passive	25 h	-	[111]
HPLC-FLD	CYCLOBOND I 2000 (25 cm × 4.6 mm I.D., 5 µm) (*Supelco, Bellefonte, PA, USA*)	Active/Passive	8 h	Activated charcoal	[112]

**Table 5:** Comparison of HPLC and GC methods for VOC analysis.

	HPLC	GC
VOCs	polar and semi-volatile	non-polar and volatile
Sample Type	Liquid	gas, air
Sensitivity	lower	higher
Detectors	UV-Vis, FLD, DAD, MS	FID, ECD, MS
Sample Prep	requires derivatisation	headspace, purge and trap, thermal desorption, or direct injection

**Table 8:** LCR values determined for indoor air around the world.

VOC	Country	LCR	Description	Ref.
Tetrachloroethylene Benzene 1,3-butadiene	China	9.35 × 10^−7^ (adults) 4.54 × 10^−4^ (adults) 1.68 × 10^−4^ (adults)	residence	[14]
1,1—dichloroethane Methylene chloride Benzene Trichloroethene Tetrachloroethene	Hong Kong	5.16 × 10^−6^ (adults) 5.14 × 10^−6^ (adults) 1.81 × 10^−5^ (adults) 1.32 × 10^−6^ (adults) 1.03 × 10^−6^ (adults)	non-smoker’s home	[148]
Benzene	Argentine	3.57 × 10^−5^ (children)	room	[147]
Benzene Formaldehyde	China	1.64 × 10^−5^ (female); 1.49 × 10^−5^ (male) 7.16 × 10^−6^ (female); 6.56 × 10^−6^ (male)	bedroom	[146]
Benzene Trichloroethylene Tetrachloroethene	India	2 × 10^−5^ (male); 4 × 10^−5^ (female) 1 × 10^−6^ (male); 3 × 10^−6^ (female) 5 × 10^−7^ (male); 1 × 10^−6^ (female)	home	[145]
Formaldehyde Acetaldehyde Benzene	Spain	7.8 × 10^−5^–4.1 × 10^−4^ (adults) 8.6 × 10^−6^–3.5 × 10^−5^ (adults) 2 × 10^−6^–1.5 × 10^−5^ (adults)	living rooms	[150]
Formaldehyde	Taiwan	1.41 × 10^−5^ (male); 1.21 × 10^−5^ (female)	renovated houses	[149]
Benzene	Turkey	5.4 × 10^−13^ (adults)	tape production facility	[151]

## Data Availability

No new data were created or analysed in this study. Data sharing is not applicable to this article.

## Appendix A

**Table A1:** Regulatory frameworks for some VOCs in countries worldwide (presented guidelines were available on the website https://www.ieqguidelines.org/table?factor=iaq (accessed on 20 April 2025).

VOCs	Levels	Environment	Time Measuring	Country	Guidelines Name
Benzene	No safe level	All building			WHO guidelines for indoor air quality: selected pollutants
	No safe level	Residential		South Africa	Guideline for the management of domestic indoor air quality
	≤0.03 mg m^−3^ No safe level 27 µg m^−3^ 10 µg m^−3^ 30 µg m^−3^	Residential	1 h acute exposure 1 year 14 days	China Canada California France	Standards for Indoor Air Quality Guidance for Benzene in Residential Indoor Air Office of Environmental Health Hazard Assessment California Environmental Protection Agency The French Agency for Food EaOHS. ANSES’s list of indoor air quality guideline values
Tetrachloroethylene	0.25 mg m^−3^	All building	1 year		WHO guidelines for indoor air quality: selected pollutants
	≤0.12 mg m^−3^		8 h	China	Standards for Indoor Air Quality
	0.25 mg m^−3^	Residential	1 year	South Africa	Guideline for the management of domestic indoor air quality
Trichloroethylene	Unit risk 4.3 × 10^–7^ per μg m^−3^	All building		South Africa	Guideline for the management of domestic indoor air quality
	≤0.006 mg m^−3^		8 h	China	Standards for Indoor Air Quality
Formaldehyde	0.1 mg m^−3^	All building	30 min		WHO guidelines for indoor air quality: selected pollutants
	120 µ m^−3^	Residential	8 h	Nigeria	National Environmental (Air Quality Control) Regulations, 2021
	≤0.08 mg m^−3^		1 h	China	Standards for Indoor Air Quality
	100 µg m^−3^	Public buildings	24 h	Thailand	Notification of indoor air quality for public buildings B.E 2565 (2022)
	100 µg m^−3^	All buildings	30 min	Japan	Committee on Sick House Syndrome: Indoor Air Pollution Progress Report No. 4—Summary of the discussions at the 8th and 9th meetings
Xylenes	≤0.20 mg m^−3^		1 h	China	Standards for Indoor Air Quality
Toluene	≤0.20 mg m^−3^		1 h	China	Standards for Indoor Air Quality
	260 µg m^−3^	All buildings		Japan	Committee on Sick House Syndrome: Indoor Air Pollution Progress Report No. 4—Summary of the discussions at the 8th and 9th meetings
Styrene	220 µg m^−3^	All buildings		Japan	Committee on Sick House Syndrome: Indoor Air Pollution Progress Report No. 4—Summary of the discussions at the 8th and 9th meetings
